# The Public Health Bill

**Published:** 1893-01

**Authors:** 


### THE PUBLIC HEALTH BILL.

The bill now before Congress, ostensibly to create a Bureau of Public Health, is suspected of harboring under a chip, a large-sized and very robust and vigorous bug, and the South will have none of it. It has aroused a degree of antagonism little suspected. The Southern States feel fully equal to the task of administering their own quarantine laws, and want no assistance from the Marine Monster, the M. H. S. The New Orleans people are thoroughly aroused on the subject, and through their Board of Health and Chamber of Commerce, have entered a strong protest against the passage of the bill; moreover, President Oliphant, of the Louisiana Board of Health, has gone to Washington, and will in person present the protest. It was drawn up by Col. Zachary, of the Chamber of Commerce, and clearly shows the unconstitutionality of the proposed step. Dr. Oliphant will be backed at Washington by representatives from other Southern States, and Dr. Jenkins, of the New York quarantine, will be there also, to defend States' rights and protest against the surrender of our State quarantines to the M. H. S. The most ridiculous feature of the bill is, that it is proposed to operate the whole thing through the Treasury Department! Why not give us a Minister of Public Health, in the Cabinet, and let each State Health Officer report to him?—leaving each State to govern its own affairs? State Health Officer Swearingen is a strong States' rights man, and is opposed to the bill now pending. He has officially expressed his views in a letter to Texas Congressman Sayers, and to the Louisiana Board of Health, who had requested it.

It is thought that large money will be expended in the effort to secure the passage of this bill in *the interest of Northern and Eastern railroad and steamship men.*

